# Differential Localization and Functional Roles of mGluR6 Paralogs in Zebrafish Retina

**DOI:** 10.1167/iovs.65.12.44

**Published:** 2024-10-30

**Authors:** Marion Haug, Sara A. Haddad-Velioglu, Manuela Berger, Anja Enz, Jingjing Zang, Stephan C F. Neuhauss

**Affiliations:** 1 University of Zurich, Department of Molecular Life Sciences, Winterthurerstrasse 190, CH-8057 Zurich, Switzerland

**Keywords:** ON-response, glutamate, zebrafish, metabotropic glutamate receptor

## Abstract

**Purpose:**

To define the location of *mglur6* paralogs in the outer zebrafish retina and delineate their contribution to retina light responses across the visual spectrum.

**Methods:**

In situ hybridization and immunolocalization with custom-made antibodies were used to localize *mglur6* transcripts, proteins, and additional components of the mGluR6 signaling complex. Gene editing was used to generate knockout mutants that were analyzed with white light and spectral electroretinography.

**Results:**

Both *mglur6* paralogs colocalized with known downstream pathway genes, such as *trpm1a*, *nyctalopin*, and *gnao*β. All rod photoreceptors contacted mGluR6-positive cells, while cone connectivity presented a more complex situation with no red cones and only a few UV and blue-sensitive cones connecting to mGluR6a-positive bipolar cells. All cone subtypes contacted mGluR6b-positive cells with markedly fewer red-sensitive cones. Retinas of knockout animals displayed no morphologic alterations. While ERG responses were unaffected in *mglur6a* knockout animals, *mglur6b* mutants displayed decreased responses over all spectral wavelengths.

**Conclusions:**

We demonstrated that mGlurR6 signalplex components are similar in the zebrafish and the mammalian retina. Despite mglur6b knockout animals having significantly impaired ERG b-wave responses, a residual b-wave persists, even in double knockouts, suggesting additional pathway components yet to be identified.

Vision starts with the sensing of light by photoreceptors in the outer retina. Subsequently, the visual stream is divided at the first visual synapse, into an ON and OFF pathway, activated by an increase or decrease of light, respectively.^1^ At the first visual synapse, on the dendritic tips of ON-bipolar cells, the metabotropic glutamate receptor 6 (mGluR6), a member of the G-protein coupled receptor family, is a central component of the ON-response.^2^

Upon binding of the neurotransmitter glutamate, mGluR6 initiates a signaling cascade, leading to the closure of TRPM1 channels and hyperpolarization of ON-bipolar cells.^3^^,^^4^ Nyctalopin, a protein enriched in dendritic tips of ON-bipolar cells, is essential for the proper localization and function of TRPM1. It interacts with TRPM1 and facilitates its trafficking to the dendritic tips, ensuring efficient signal transduction in the retina.^5^^,^^6^ A light-initiated decrease in glutamate release leads to the deactivation of mGluR6 and the opening of TRPM1 channels, causing depolarization of ON-bipolar cells.^6^ The pre- and postsynaptic molecules and downstream effectors that contribute to mGluR6 mediated ON-signaling have been termed the signalplex (for reviews, see Martemyanov and Sampath^2^ and Furukawa et al.^7^). The significance of this signaling cascade for human vision is further demonstrated by the association of congenital stationary night blindness of the Schubert–Bornschein type with mutations in various components of the transduction cascade (reviewed in Zeitz et al.^8^).

Many of the signalplex molecular components have been described in vertebrate species, most extensively in nocturnal mice. Diurnal zebrafish possess a cone-dominated retina, making them particularly suitable for studying cone-mediated visual functions. In a previous study, we identified two paralogs of *mglur6* that are both expressed in the retina and showed that the knockdown of one of them (*mglur6b*) led to a diminished Schubert–Bornstein type electroretinogram (ERG).^9^ In this study, we set out to investigate the localization of the mGluR6 proteins in relation to rod and cone synapses. Using paralog-specific antibodies and transgenic lines, we localized both mGluR6 proteins to all rod-contacting synapses. Moreover, mGluR6b proteins are localized to all four types of cone synapses, with markedly fewer red cone synapses, while mGluR6a protein mainly localizes to green cone synapses, as well as few UV and blue cone synapses, and are absent from red cone synapses. Additionally, we showed that the main components of the signalplex are present and coexpressed within the same cells in the zebrafish retina using fluorescence in situ hybridization (FISH). Finally, we generated knockout strains for the two mglur6 paralogs for ERG analysis. While no functional impairments in knockout animals of *mglur6a* were apparent, we demonstrated that the mGluR6b paralog is essential for normal b-wave responses to visual stimuli encompassing a range of wavelength across the light spectrum.

## Materials and Methods

### Fish Maintenance and Breeding

Zebrafish (*Danio rerio*, Tü and WIK wild-type strains) were kept under standard conditions at a 14-hour/10-hour light/dark cycle at 26°C as described.^10^ Embryos were raised at 28°C in E3 medium and staged according to development in days postfertilization (dpf).^11^ Research was performed in accordance with the ARVO Statement for the Use of Animals in Ophthalmic and Vision Research and was approved by the local authorities (Veterinäramt Zürich TV4206).

### FISH

Fragments of the *mglur6a*, *mglur6b*, *gnaoa*, *trpm1a*, and *nyx* coding sequence were amplified from 5 dpf zebrafish cDNA with primers listed in Supplementary Table S1. FISH was performed as described in Haug et al.^12^

### Immunohistochemistry, Antibody Generation, and Standard Histology

Polyclonal antibodies were generated (epitopes CKSINEKQNGETKIEPDRTQ (mGluR6a), QKSSDKQNGETKVEPDRSQ (mGluR6b) and specificity established as described for mGluR6a^13^ and mGluR6b.^9^ Immunohistochemistry was performed as described in Huang et al.^9^ Standard histology on plastic sections were performed as described in Haug et al.^14^

### CRISPR/Cas9 Mutagenesis

Crispr/Cas9 mutagenesis was performed as described elsewhere.^15^^–^^17^ CRISPR target site formglur6a and mglur6b sequences were selected, and oligonucleotides for cloning single-guide RNA (sgRNA) templates (Supplementary Table S1) were retrieved using CHOPCHOP (chopchop.rc.fas.harvard.edu). Forward and reverse oligonucleotides were cloned into pT7- or pSP6-sgRNA as described in Jao et al.^18^ and amplified using primers listed in Supplementary Table S1. sgRNAs were transcribed using the Megashortscript T7 kit (Invitrogen, Carlsbad, CA, USA), the Megascript Sp6 kit (Ambion, Austin, TX, USA), or the Megashortscript T7 kit (Ambion) and purified using isopropanol and ammonium acetate with the following protocol^19^ or purified using the MEGAclear kit (Ambion).

Adult F0 fish were outcrossed with wild-type fish, and F1 offspring were analyzed for germline transmission by genotyping individual fin biopsies. Targeted regions of *mglur6a* and *mglur6b* were amplified (primers; see Supplementary Table S1), cloned into pCRII-TOPO (Invitrogen), and subsequently sequenced. F1 fish with identical heterozygous frameshift mutations were crossed, and the F2 generation was genotyped and analyzed by gel electrophoresis.

The mutagenesis caused a deletion of 103 base pairs in the locus of *mglur6a* and an indel of +27/–7 base pairs in the *mglur6b* locus, both leading to a frameshift and a nonfunctional protein (see Supplementary Fig. S3 for the mutations).

### ERG Measurements

Single flash electroretinogram recordings on 5 dpf larvae were performed as described in Zang et al.^20^ Stimulation for spectral ERG measurements was performed as previously described on 5-day-old larvae and 1-month-old juvenile zebrafish in Zang et al.^20^ More specifically, a Xenon light source (HPX-2000; Ocean Optics, Orlando, FL, USA), combined with various filters, was used as the stimulation light source. A LED light source (Ocean Optics, LSM series) with different filters served as the background light source to adapt photoreceptors not of interest. For red light stimulation, wavelengths above 570 nm were used without background light. For green light stimulation, light at 472 nm was used, with wavelengths above 570 nm as the background to adapt red cones. For blue and UV light stimulation, wavelengths below 420 nm were used as the stimulation light, while light above 470 nm was used as the background. The amplitude of the ERG signal was extracted using Excel (16.1.3; Microsoft, Redmond, WA, USA). The b-wave amplitude was calculated from the trough of the a-wave to the peak of the b-wave.^21^ Data analysis and preparation of graphs was performed in GraphPad Prism (8.4; GraphPad Software, La Jolla, CA, USA). One-month-old zebrafish recordings were collected as described above, but prior to isolation of the eye, fish were euthanized with MESAB in E3 in accordance with Fachinformation 3.01.

Data were analyzed using GraphPad Prism version 10.1.2 for Windows (GraphPad Software), where we performed a two-way ANOVA using a mixed-effects model with a Geisser–Greenhouse correction and a Tukey's multiple comparison test.

## Results

### Cellular Coexpression of mRNAs Encoding Elements of the mGluR6 Signaling Complex

To establish the presence of mglur6 paralogs and the main components of the mGluR6 signaling complex, such as Goa, TrpM1 channels, and nyctalopin, in the zebrafish retina, we performed double-fluorescent in situ hybridization with riboprobes specific for the mRNAs of these molecules. Fluorescent signals were primarily observed in the nuclei, with occasional labeling in synaptic regions. Colocalization was not strictly necessary for transcripts expressed within the same cell, but labeling often appeared in close proximity.

**Figure 1.**
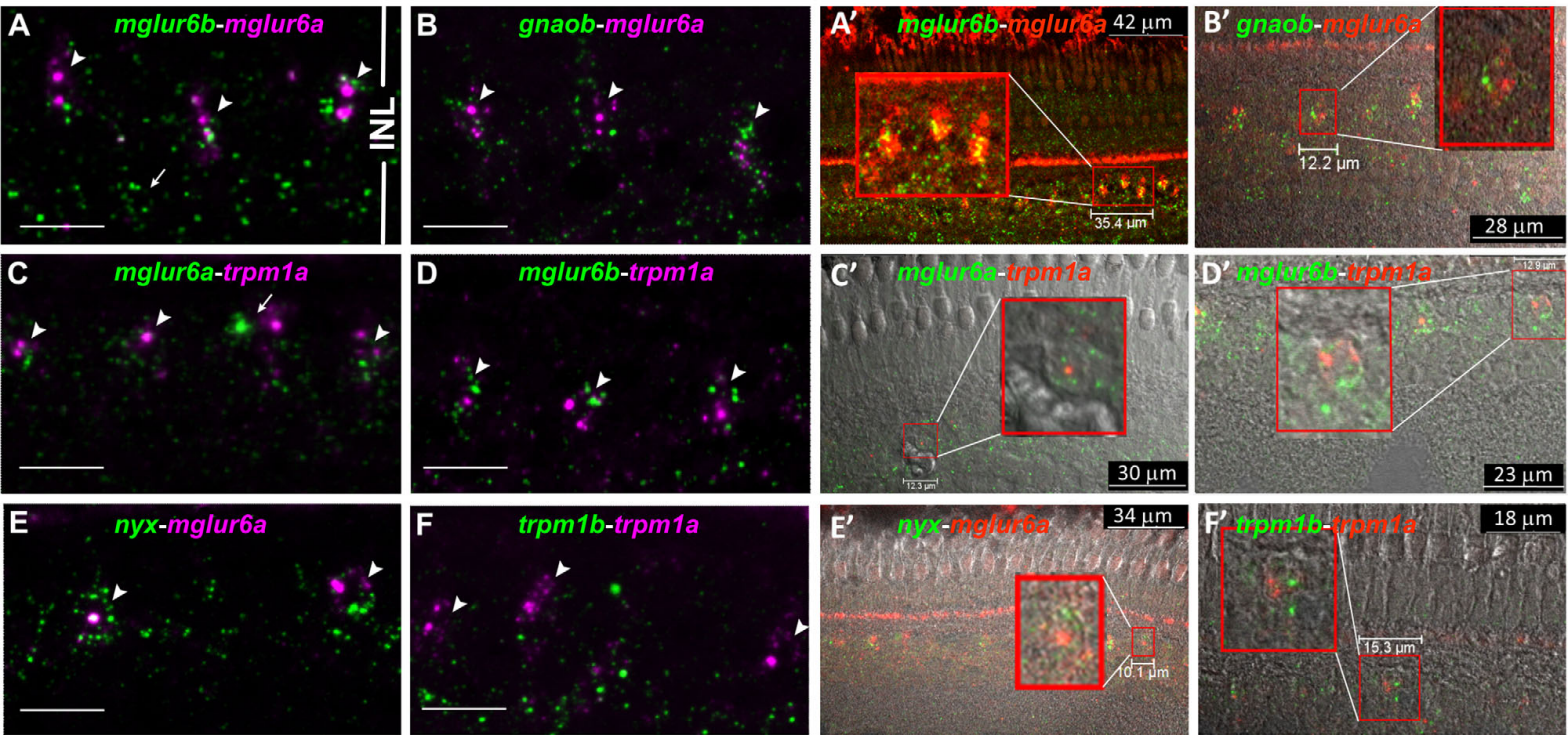
Localization of *mglur6*-pathway genes labeled by fluorescence in situ hybridization. (**A–F**) Z-stacks of two to three images of adult zebrafish retinal slices taken with a confocal microscope. (**A′–F**′) Genes in A–F are coexpressed in the same cell body as shown by differential interference contrast (DIC) images as background. (**A**, **A**′) The *arrowheads* point to bipolar cell soma where *mglur6b* and *mglur6a* colocalize. While *mglur6a* labeling is restricted to the medial inner nuclear layer (INL), the *mglur6b* riboprobe additionally labels cell bodies in the proximal INL (*arrows*). (**B**, **B**′) The α-subunit of the G-protein (*gnaob*) is located in the same ON-bipolar cells as *mglur6a* (*arrowheads*). (**C**, **C**′) *mglur6a* colocalizes with *trpm1a* in ON-bipolar cells (*arrowheads*), but for some cell bodies, it is unclear whether exactly the same soma or two different cell bodies next to each other are labeled (*arrow*). (**D**, **D**′) The *mglur6b* riboprobe is expressed in the same cells where *trpm1a* is located (*arrowheads*). (**E**, **E**′) The cation channel *trpm1a* colocalizes with *nyx* in green in the medial INL (*arrowheads*). (**F**, **F**′) Both *trpm1* channel genes are expressed in the INL. While *trpm1a* clearly labels ON-bipolar cell soma (*arrowheads*), *trpm1b* is only marginally expressed in the medial INL but rather labels structures in the proximal INL. All images reveal the whole INL, as depicted in **D**. *Scale bars*: 10 µm. *Scale bars* differ in each figure, so they are individually labeled.

We found that *gnaob*, *trpm1a*, *trpm1b*, *nyx*, and both mglur6 paralogs were expressed in close apposition within the inner nuclear layer (Figs. 1A–F). High-resolution images revealed that each of these components and at least one of the *mglur6* paralogs were frequently present within the same cell bodies, as confirmed by bright-field microscopy (Figs. 1A–F). These findings suggest that the mGluR6 signaling complex components are conserved and present in the zebrafish retina, indicating a likely conserved function.

### mGluR6 Paralog Expression in Larval and Adult Retina Tissue

We next examined the subcellular localization of the mGluR6 paralogous proteins in the retina with custom-generated paralog-specific antibodies throughout development.

**Figure 2.**
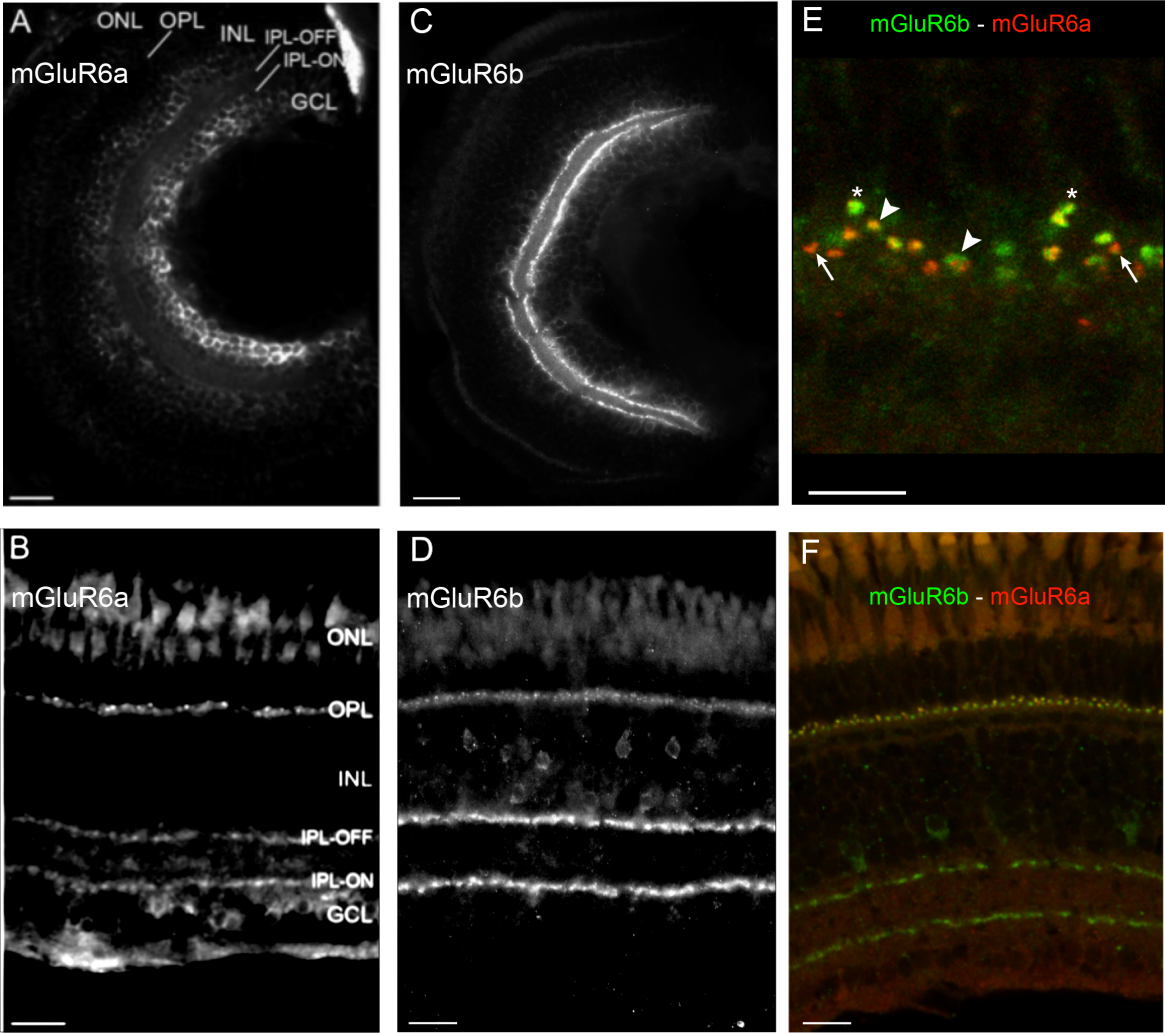
Retinal expression of mGluR6 paralogs in larval and adult zebrafish. (**A–D**) Immunohistochemical labeling of mGluR6a on larval (**A**) and adult (**B**) and mGluR6b on larval (**C**) and adult (**D**) retinal cross sections. (**E**, **F**) Costaining of mGluR6a (*red*) and mGluR6b (*green*) on adult retinal cross sections. In (**E**), a zoom-in on the OPL shows where mGluR6b (*asterisk*) and mGluR6a (*long arrow*) do not overlap and where mGluR6a and mGluR6b expression overlaps (*large arrowhead*) (see Supplementary Table S2). GCL, ganglion cell layer; INL, inner nuclear layer; IPL‐OFF, off‐layer of the inner plexiform layer; IPL‐ON, on‐layer of the inner plexiform layer; ONL, outer nuclear layer; OPL, outer plexiform layer. *Scale bars*: 20 µm, except **E**, where the *scale bar* = 5 µm.

At 5 dpf, we found that mGluR6a protein was expressed diffusely around cell bodies in the deeper retinal layers but not in the outer plexiform layer (Fig. 2A), whereas mGluR6b was expressed in both the outer and inner plexiform layers (Fig. 2C). In adult tissue, both mGluR6 paralogs were expressed in the outer and inner plexiform layers (Figs. 2B, 2D). When we examined adult retinal tissue with higher magnification, we observed mGluR6 paralog coexpression in puncta found in the outer plexiform layer. mGluR6a and mGluR6b occasionally colocalized (large arrowheads) and sometimes were localized specifically in different puncta (asterisks show mGluR6b-positive puncta, narrow arrows show mGluR6a-positive puncta) (Fig. 2E).

### mGluR6 Paralogs Localized to Specific Cone Photoreceptor Subtypes

As an initial step to define the paralog-specific functions of mGluR6 proteins, we examined the coexpression of the two mGluR6 using paralog-specific antibodies with various photoreceptor types using transgenic lines that label distinct photoreceptor subtypes in the adult retina.^22^^–^^26^

**Figure 3.**
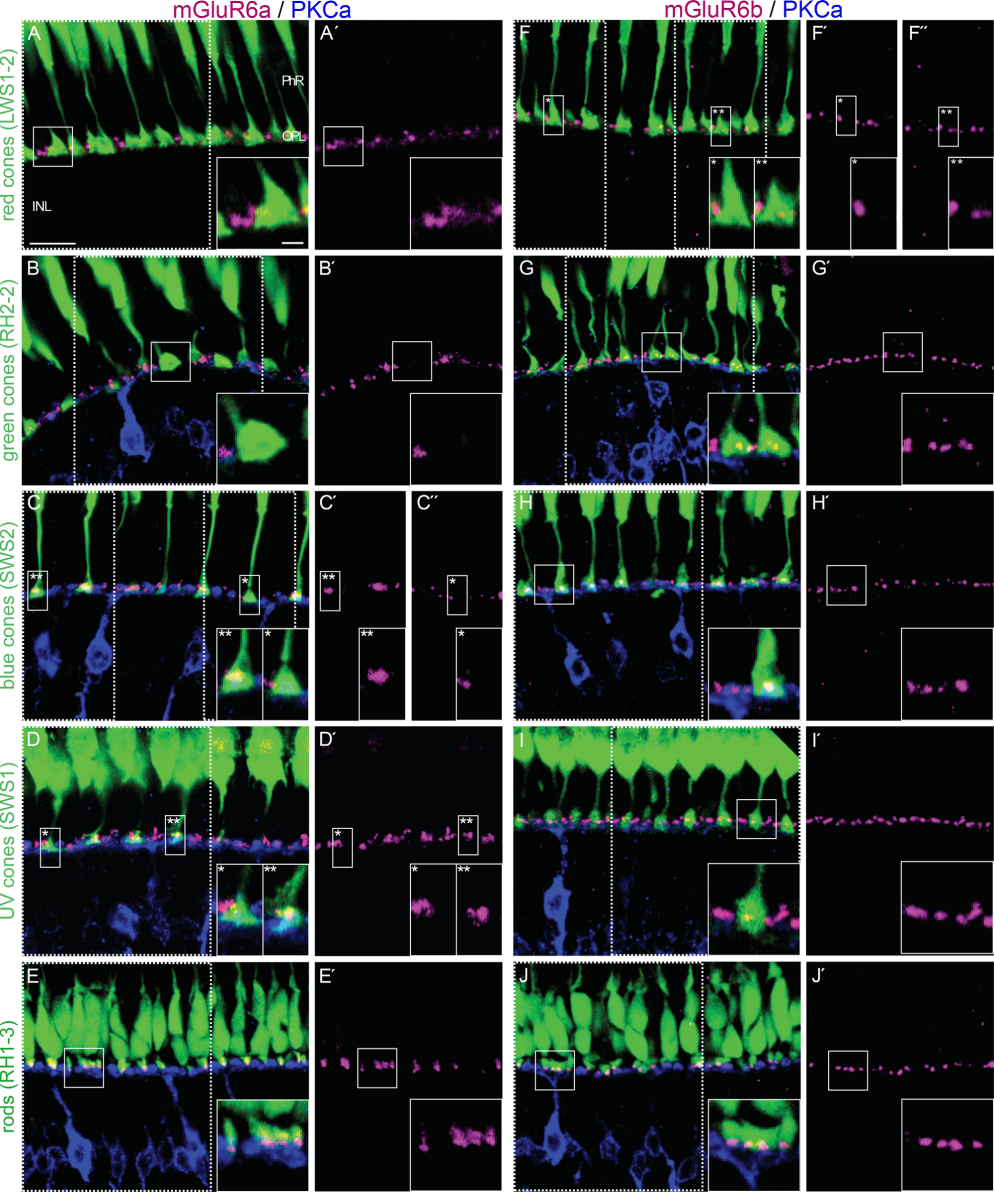
mGluR6a paralog expression in photoreceptor subtype synapses. (**A–D**′) Z-stacks of adult retinal slices taken with a confocal microscope. The different transgenic lines express GFP under different cone opsin promotors. Anti-PKCα antibody staining labels ON-bipolar cells in *blue*; in panels **A–E**′, pink labels mGluR6a puncta; and in panels **F–J**′, pink labels mGluR6b puncta. (**A, A**′**)** LWS1-2 transgenic fish are expressing GFP in their red cones. (**B, B**′) RH2-2 transgenic fish expressing GFP in green cones. (**C, C**′) SWS2 transgenic fish expressing GFP in blue cones (*mGluR6a negative; **mGluR6a positive). (**D, D**′) SWS1 transgenic fish expressing GFP in their UV cones. (**E, E**′) RH1-3 transgenic fish line expressing GFP in rod photoreceptors. (**F, F**′) LWS1-2 transgenic fish expressing GFP in their red cones (*mGluR6b negative; **mGluR6b positive). (**G, G**′) RH2-2 transgenic fish expressing GFP in green cones. (**H, H**′) SWS2 transgenic fish expressing GFP in their blue cones. (**I, I**′) SWS1 transgenic fish expressing GFP in their UV cones. (**J, J**′) RH1-3 transgenic fish line expressing GFP in their rod photoreceptors. The closeups in every image show representative cone-to-ON-bipolar cell synapses for the corresponding staining. *Scale bar*: 10 µm; *scale bar* of the closeup: 2 µm.

We did not detect mGluR6a in synapses of red cones (Fig. 3A) but found them to be restricted to subsets of synapses of green cones (more than half), blue cones (less than half), and few UV cones (Figs. 3B–D). However, mGluR6a was present in all rod photoreceptor synapses (Fig. 3E). mGluR6b protein localized to most red cone photoreceptors (Fig. 3F) and all green, blue, UV cone, and rod photoreceptors (Figs. 3G–J). Intriguingly, some red cone photoreceptor synapses did not contain detectable immunofluorescence of either mGluR6a or mGluR6b. These data are based on a section of between two and six eyes and between 16 and 180 cell counts.

### mGluR6a-Depleted Fish Showed Normal ERG Responses, Whereas mGluR6b Mutants Showed Largely Diminished ERGs Across the Whole Light Spectrum

To further investigate the functional significance of the mGluR6 paralogs, we generated knockout lines for each paralog using the Crispr/Cas9 system. First, we confirmed that there were no obvious morphologic differences present in mutant larval (5 dpf) and adult retinas using standard histology on plastic sections (Supplementary Figs. S4A–H). Additionally, we confirmed the gene deletion using antibody staining to verify the absence of expression at 5 dpf, while staining of the corresponding paralog was unaltered (Supplementary Figs. S4I–N).

**Figure 4.**
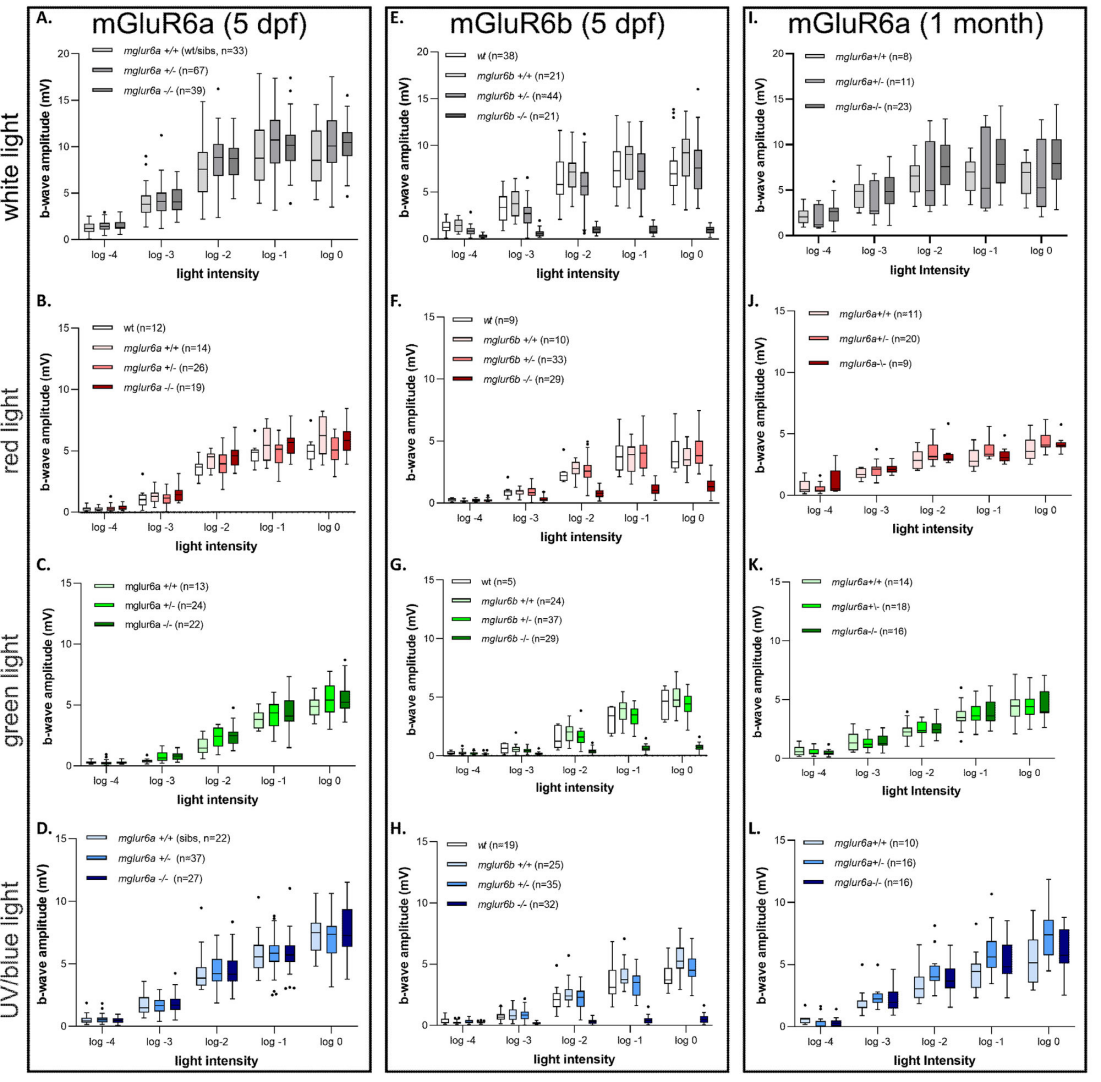
Spectral ERG measurements of the peak b-wave response in *mglur6a* and *mglur6b* mutant fish. The peak b-wave amplitude was quantified at five different light intensity stimuli, at different spectral wavelengths. (**A–D**) The peak b-wave measured in 5 dpf *mglur6a* mutant fish. There is no significant difference between wild-type and KO fish in any of the various spectra (see Materials and Methods for wavelengths of various stimuli). (**A**) White light stimulus. (**B**) Red light stimulus. (**C**) Green light stimulus. (**D**) UV/blue light stimulus. (**E–H**) The peak b-wave measured in 5 dpf *mglur6b* mutant fish. *mglur6b^–^^/^^–^* fish show a significantly decreased b-wave amplitude in response to all stimuli. (**E**) White light stimulus. (**F**) Red light stimulus. (**G**) Green light stimulus. (**H**) UV/blue light stimulus. (**I–L**) The peak b-wave measured in adult mGluR6a mutant fish. There is no significant difference between wild-type and KO fish in any of the various spectra. (**I**) White light stimulus. (**J**) Red light stimulus. (**K**) Green light stimulus. (**L**) UV/blue light stimulus. (For all statistical data and significance, please see Supplementary Table S3.)

For functional assessment of the outer retina, we first performed ERG recordings using different light spectrum stimuli in 5 dpf larvae for both *mglur6* mutant strains. We found no difference in the ERG response for any of the light stimuli in *mglur6a* knockout (KO) at 5 dpf (Figs. 4A–D). In contrast to and consistent with previous results from knockdown animals,^9^ we found that the ERG response was greatly diminished across all spectra in *mglur6b* KO fish at 5 dpf (Figs. 4E–H). Remarkably, we were unable to completely abolish the b-wave response, as a small residual b-wave at higher stimulus intensities across all stimuli spectra remained (Figs. 4E–H). Interestingly, the least diminished b-wave in the *mglur6b* KO fish was in the red spectra, which aligns with our observation that mGluR6b was not present in all red cones (Fig. 3F and Supplementary Table S1).

To address the unexpected results of the *mglur6a* KO fish, we repeated the ERG analysis on fully developed adult retinas. Consistent with the 5 dpf results, we found no difference in the ERG response in older zebrafish in any of the light spectra we tested (Figs. 4I–L). We then examined the kinetics of the ERG b-wave in *mglur6a* mutants at both 5 dpf (Supplementary Fig. S5) and adult stage (Supplementary Fig. S6). The time to b-wave peak, exponential decay time constant, and decay half-life were quantified. We did not observe apparent defects in the mutants, indicating that mGlur6a may not shape the overall ON response kinetics.

Since we identified the presence of mGluR6a in the outer retina, this surprising result suggests an unidentified role for mGluR6a in zebrafish outer retina function.

**Figure 5.**
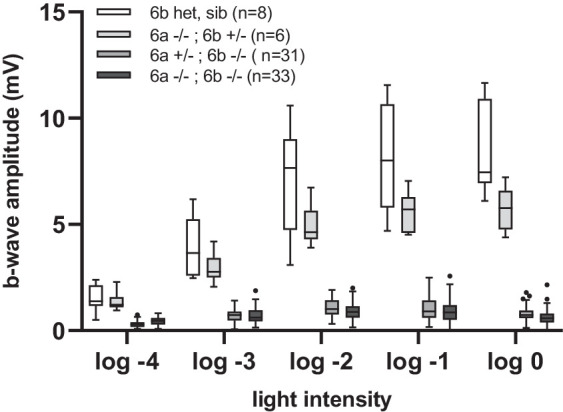
White light ERG measurements of the peak b-wave response in *mglur6* paralog double-mutant fish. The peak b-wave amplitude was quantified at 5 different light intensity stimuli, using a white light stimulus, in double-mutant *mglur6a^–^^/^^–^* *mglur6b^–^^/^^–^* fish in comparison to heterozygous crosses and controls. (For all statistical data and significance, see Supplementary Excel Sheet 1.)

Finally, we performed white light ERG measurements on double-mutant fish, depleted of both paralogs at larval stages. As expected, we measured a large reduction of the b-wave, similar to the recordings in single *mglur6b* knockout larvae (Fig. 5). Consistent with our previous findings, there was still a detectable b-wave at higher stimulus intensities in the double KO fish. The response kinetics in *mglur6a* mutants in *mglur6b* heterozygous and wild-type backgrounds remained very similar to the controls (Supplementary Fig. S7).

## Discussion

In the vertebrate central nervous system, glutamate mainly acts as an excitatory neurotransmitter. However, in the visual system, it serves an inhibitory role at the synapse between photoreceptors and ON-bipolar cells, facilitating light-ON signal inversion by stopping the “dark current.” This involves the mGluR6 receptor and its signaling complex, including TRPM1, nyctalopin, and Gαo proteins.^5^^,^^6^^,^^27^^,^^28^

The current as well as previous studies from our group showed coexpression of all studied members of the transduction cascade in the inner retina.^9^^,^^29^^–^^31^

As a result of a genome duplication event in the teleost lineage,^32^ the zebrafish genome contains two *mglur6* paralogs, *mglur6a* and *mglur6b*. The retention of both paralogs suggests potential functional divergence. To investigate their protein localization, we generated paralog-specific antibodies for subcellular immunolabeling. Consistent with our previous findings,^9^ both paralogs are expressed in the inner nuclear as well as the ganglion cell layer of the retina. Given that metabotropic glutamate receptors can form functional heterodimers,^33^^,^^34^ we assessed protein coexpression. As anticipated, both proteins were observed at dendritic tips of bipolar cells in the outer plexiform layer, with occasional overlap, opening the possibility of heterodimer formation.

We further explored whether the two paralogs might be involved in processing different spectral information of light. Using different transgenic opsin lines to label rod and cone synapses, we detected distinct expression patterns. The mGluR6a protein was primarily localized in green cone and some UV and blue cone synapses but was absent from red cone synapses. In contrast, the mGluR6b protein was present across all cone synapse types but with fewer contacts in red cone synapses. Both proteins were found in rod pedicles.

In a previous study, we reported a reduction in the ERG b-wave following downregulation of the mGluR6b protein using morpholino antisense technology.^9^ In the present study, we generated knockout lines for both paralogs using CRISPR-Cas9 genome editing, allowing us to establish stable lines and perform functional experiments at postlarval stages. We confirmed that the mGluR6b protein is involved in the ON-response under both white and spectral light stimulation, with a slightly less pronounced attenuation under red light.

Unexpectedly, mGluR6a-deficient fish exhibited largely normal light responses in the ERG. Thus, the function of mGluR6a in the retina remains unclear. Considering that ERG predominantly reflects outer retinal function, it is possible that mGluR6a plays a role in ganglion cells or has subtle functions in the outer retina that are not captured by the summed field recordings of the ERG.

In ray-finned fishes, a distinctive glutamate-driven cascade has been proposed, where cone photoreceptor ON-signaling is mediated by excitatory amino acid transporters (EAATs), distinguishing it from the mGluR6-centric pathway primarily implicated in rod to ON-bipolar cell communication.^35^^–^^39^ EAATs are ideally poised to mediate hyperpolarization upon glutamate binding, since as bifunctional molecules, they induce an uncoupled chloride conductance upon glutamate binding.^40^^,^^41^

This study and two other recent studies^41^^,^^42^ dispel the previous assumptions about cone-to-ON-bipolar cell signaling being predominantly mediated by EAATs in the zebrafish retina. Nevertheless, the small remaining b-wave detectable in *mglur6a/b* double mutants suggests a measurable but minor contribution by the EAAT signaling pathway.^41^^,^^42^

In particular, the absence of evidence for a role of mGluR6a in the generation of the ON-response implies yet unknown contributions of mGluR6 signaling to retina and brain function. This is further supported by the observation that mGluR6, once believed to be exclusively expressed on ON-bipolar cells, is expressed outside the outer retina^9^^,^^31^^,^^43^ and in various brain regions.^31^^,^^43^

In conclusion, we could show that similar to mammals, rod and cone ON-responses are mainly driven by a mGluR6b signaling complex. However, the role of the second paralogous protein mGluR6a remains elusive. Moreover, at least in teleosts, there is also a minor contribution of EAAT proteins (EAAT5b, EAAT7) that function as glutamate transporters and glutamate gated chloride channels.^41^^,^^42^

## Supplementary Material

Supplement 1

Supplement 2

Supplement 3

Supplement 4

Supplement 5

Supplement 6

Supplement 7

Supplement 8

Supplement 9
