# Supplementary material for: Differential Localization and Functional Roles of mGluR6 Paralogs in Zebrafish Retina
Source: Invest Ophthalmol Vis Sci. 2024 Oct 30;65(12):44. doi: 10.1167/iovs.65.12.44 (PMC11536201; doi:10.1167/iovs.65.12.44)
Supplement: Supplement 1 [file iovs-65-12-44_s001.pdf]

mglur6a

wt:
GTCCTGGAGGCCGCGAGGAGGTCCAATCTAACCGGTCATTTTAAGTTTGTGGGATCAGACAG
CTGGGGTGCCAAGAGCTCCCCGATTCTGGACCAGGAGGACGTGGCTGAAGGCGCCGTCACC

mut:
GTCCTGGAGGCCGCGAGGAGGTCCAATCTAACCGGTCATTTTAAGTTTGTGGGATCAGACAG
CTGGGGTGCCAAGAGCTCCCCGATTCTGGACCAGGAGGACGTGGCTGAAGGCGCCGTCACC -103 bp

mglur6b

wt: AAAGGCTAATGGAGACATCCAATGCCAGAGGGGTTATTATTTT

mut: AAAGGCTAATGGAGACATCCAGACATATTGGCAATGTTTAGACATCCAAGGGGTTATTATTTT +27/-7: +20 bp

Supplemental image S3: Location of mutation and resulting indels generating the frame shift mutations for the *mGlur6* paralog knockout fish. For *mglur6b* the region is in exon 3 and for *mglur6a* the region is in exon 4.
