# Supplementary material for: Differential Localization and Functional Roles of mGluR6 Paralogs in Zebrafish Retina
Source: Invest Ophthalmol Vis Sci. 2024 Oct 30;65(12):44. doi: 10.1167/iovs.65.12.44 (PMC11536201; doi:10.1167/iovs.65.12.44)
Supplement: Supplement 2 [file iovs-65-12-44_s002.pdf]

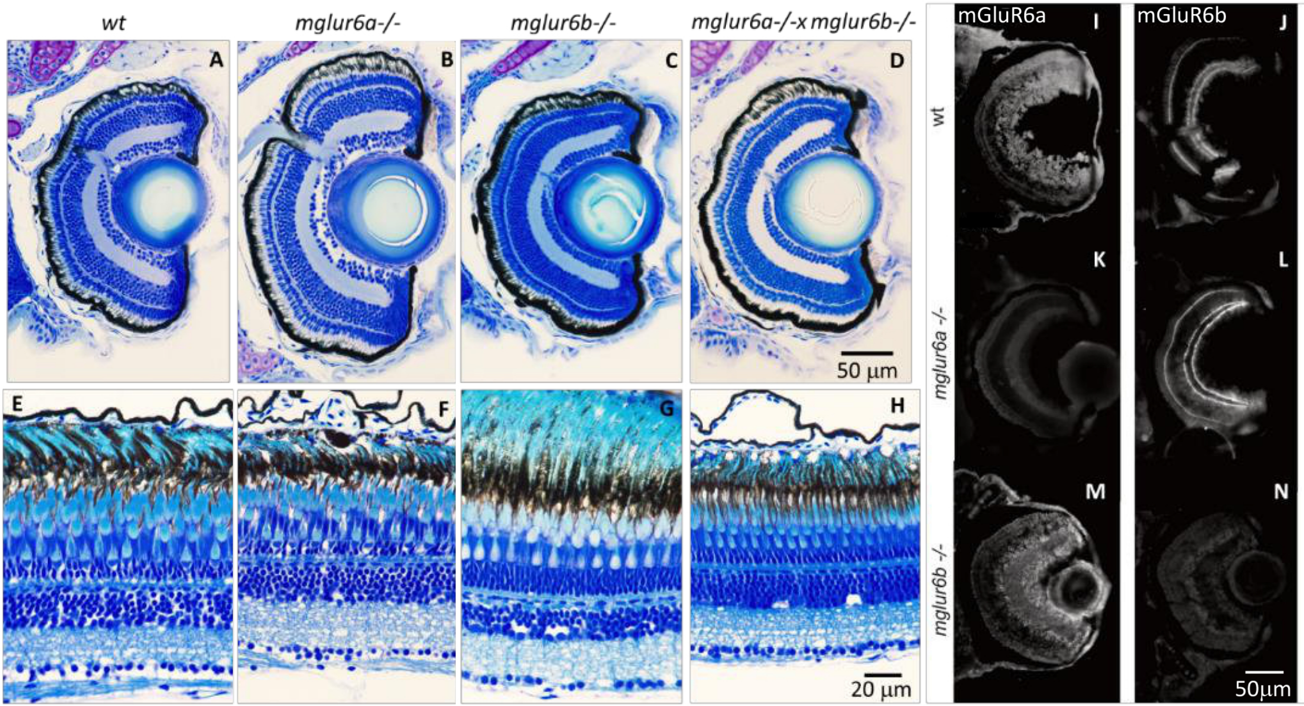


**Supplemental image S4**: Richardson staining of Technovit plastic sections and IHC staining showing morphology and KO confirmation, supplement to Figure 4. A-H. Richardson staining of Technovit plastic sections of 5dpf larval and adult zebrafish eyes of wildtype and mutant fish show no apparent morphological differences. A-D 5dpf larval eye sections, Scale bar = 50 µm. (A) wt fish (B) mGlur6a-/- mutant fish (C) mGlur6b-/- mutant fish (D) mGlur6a-/- x mGlur6b-/- mutant fish. E-H Adult eye sections, Scale bar = 20 µm. (E) wt fish (F) mGlur6a-/- mutant fish (G) mGlur6b-/- mutant fish (H) mGlur6a-/- x mGlur6b-/- mutant fish. I-N. IHC staining of 5dpf larval eye sections with mGlur6 paralog antibodies in wt and mutant fish show successful knock-out of paralogs and no differences in the none-Knocked out paralog staining, Scale bar = 50 µm. (I) wt tissue with mGlur6a antibody staining (green). (J) wt tissue with mGlur6b antibody staining. (K) mGlur6a-/- mutant tissue with mGlur6a antibody staining. (L) mGlur6a-/- mutant tissue with mGlur6b antibody staining. (M) mGlur6b-/- mutant tissue with mGlur6a antibody staining. (N) mGlur6b-/- mutant tissue with mGlur6b antibody staining. Scale bar = 50 µm.
