# Supplementary figures and images for: Differential Localization and Functional Roles of mGluR6 Paralogs in Zebrafish Retina
Source: Invest Ophthalmol Vis Sci. 2024 Oct 30;65(12):44. doi: 10.1167/iovs.65.12.44 (PMC11536201; doi:10.1167/iovs.65.12.44)

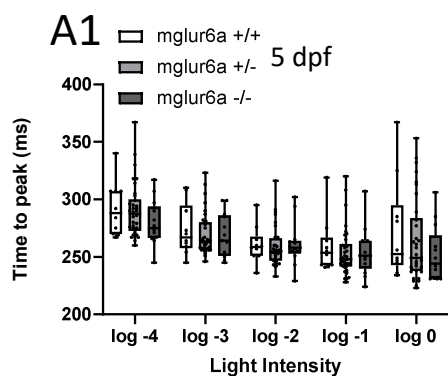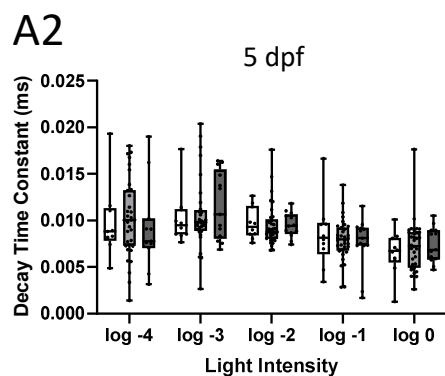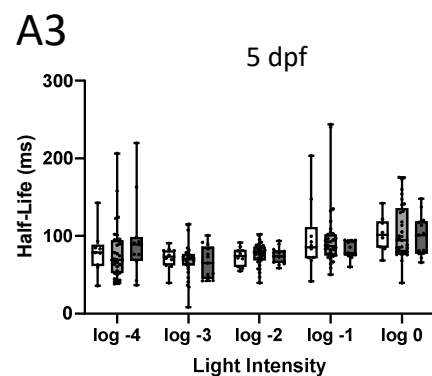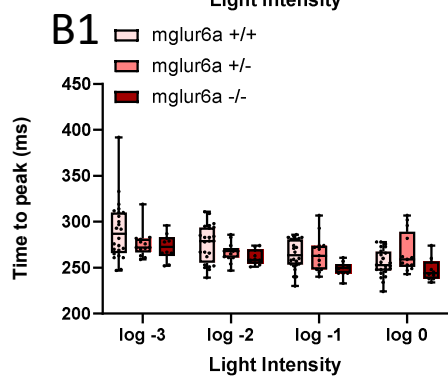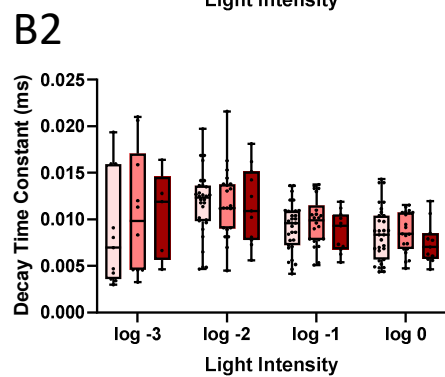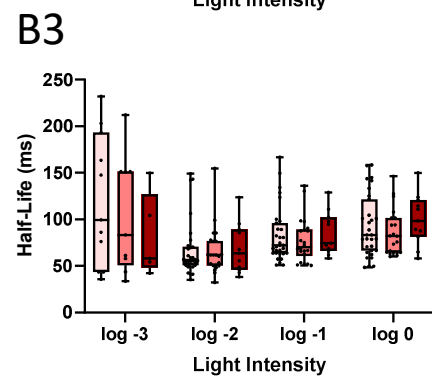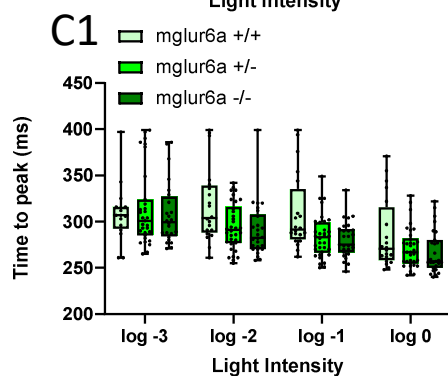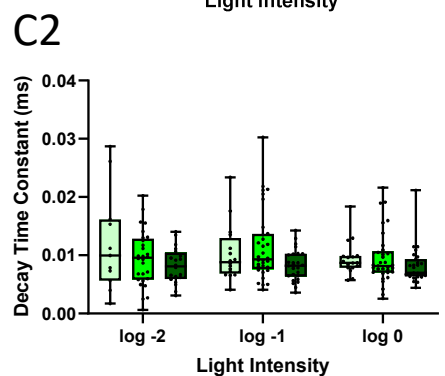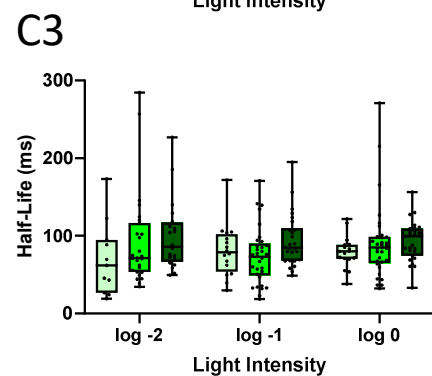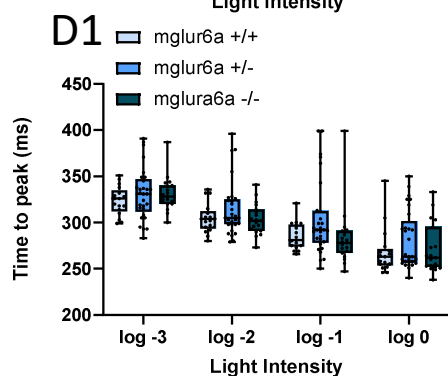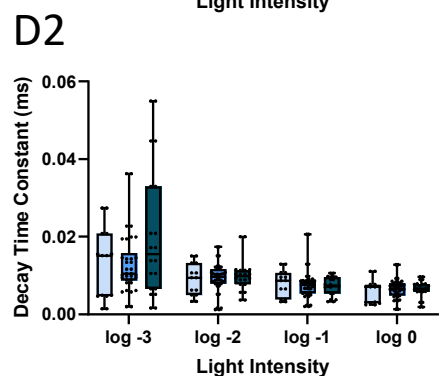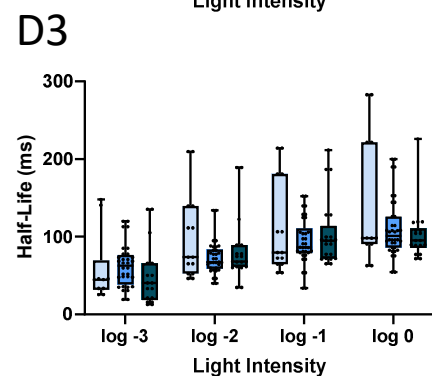

Supplement: Supplement 3 [file iovs-65-12-44_s003.pdf]

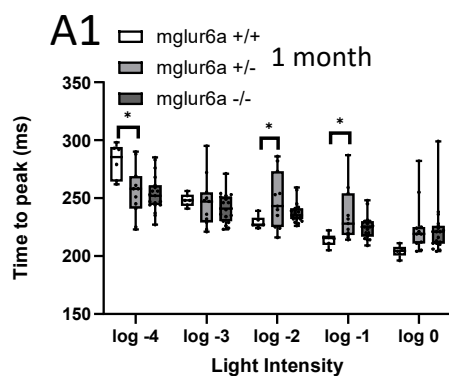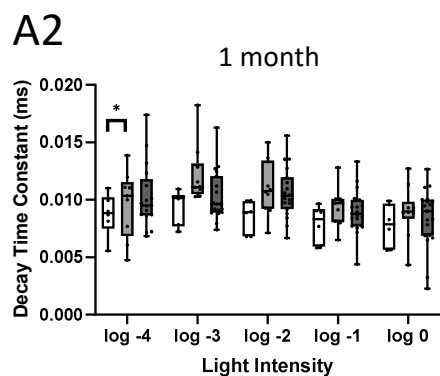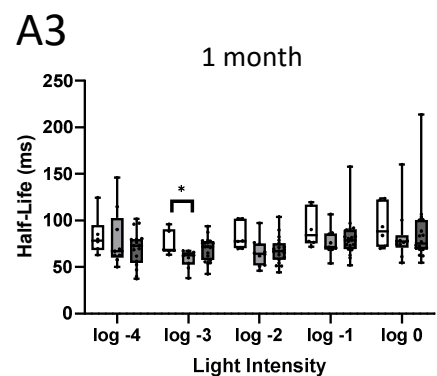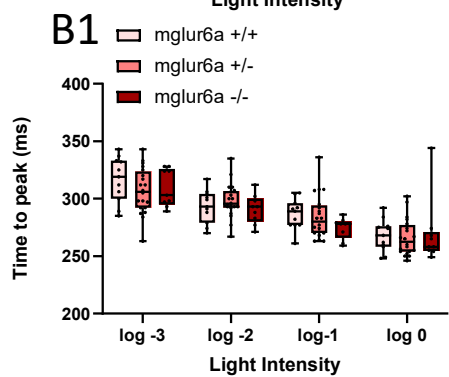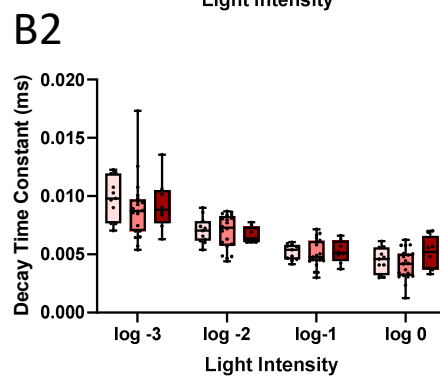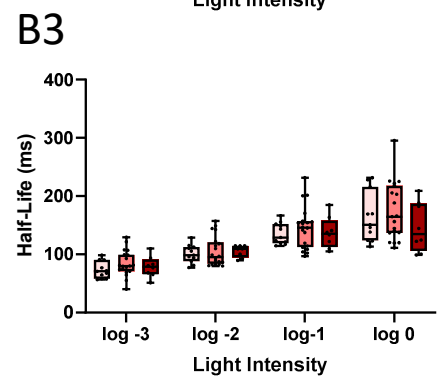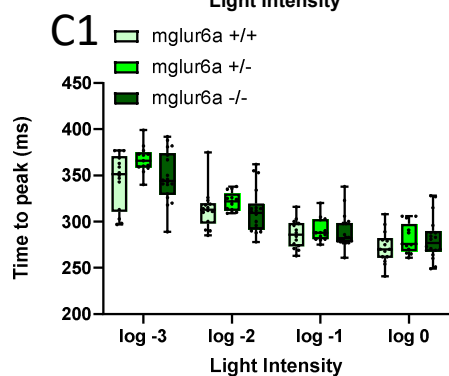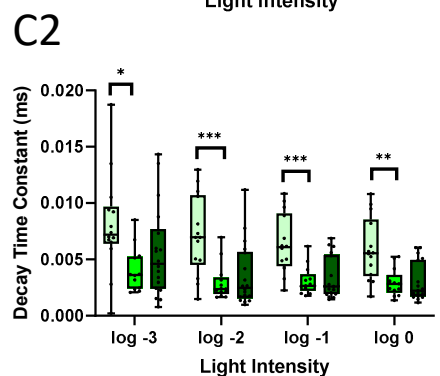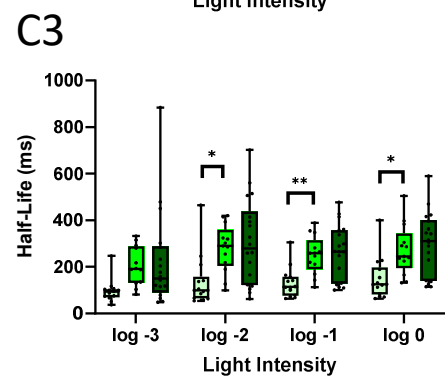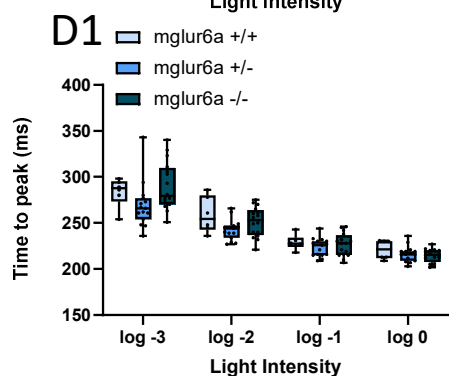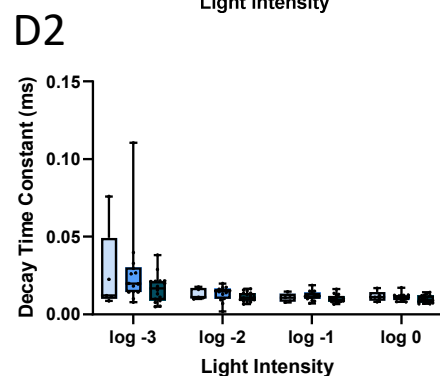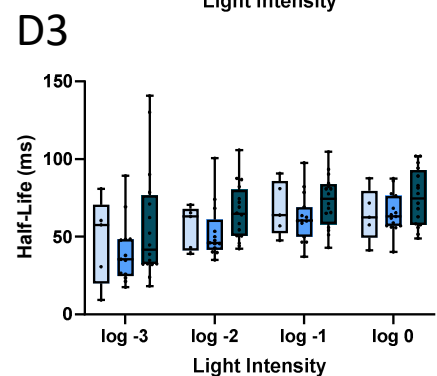

Supplement: Supplement 4 [file iovs-65-12-44_s004.pdf]

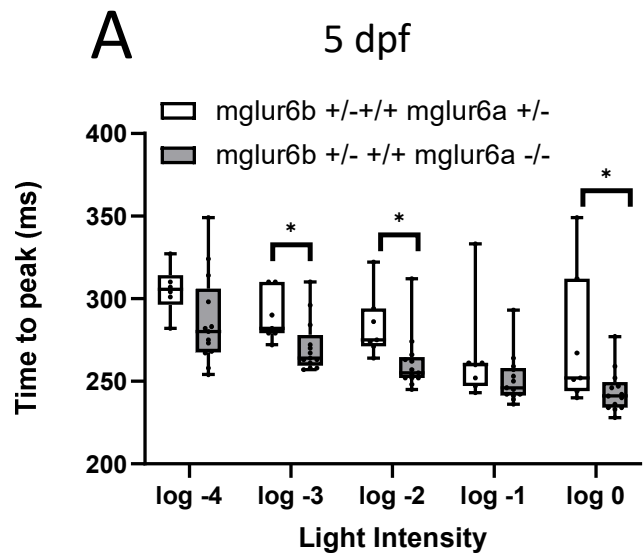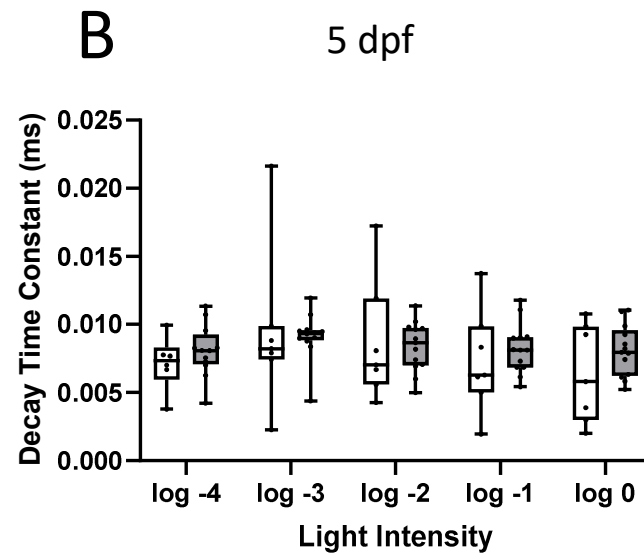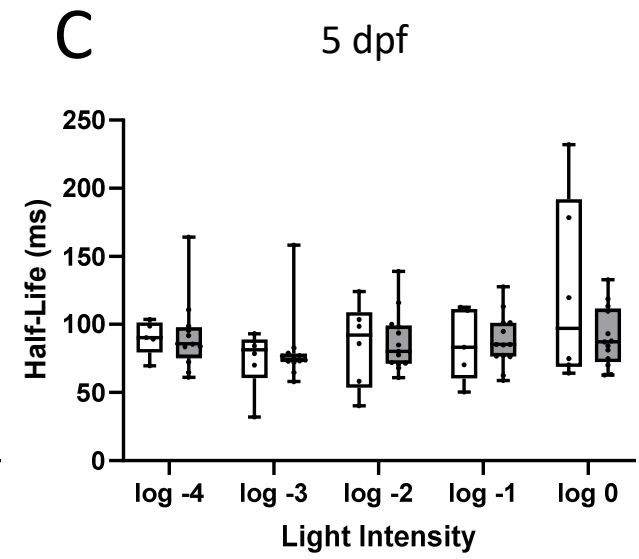

Supplement: Supplement 5 [file iovs-65-12-44_s005.pdf]

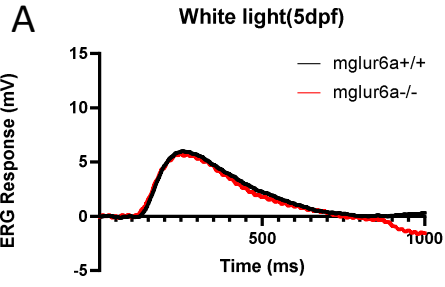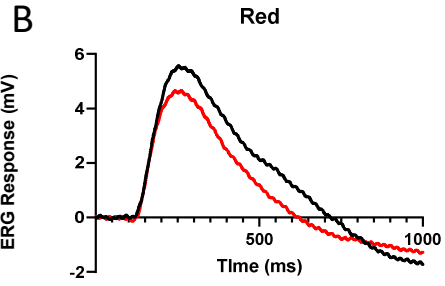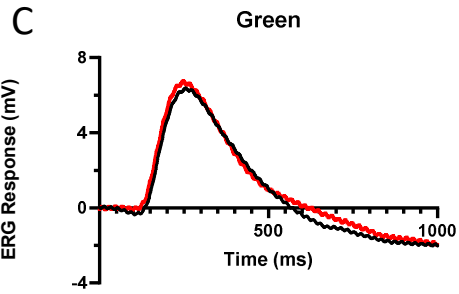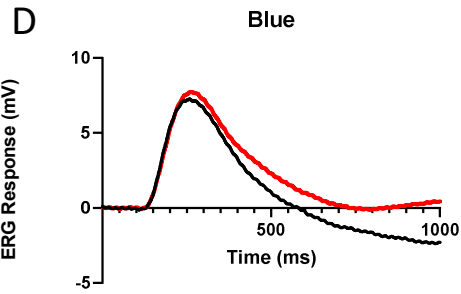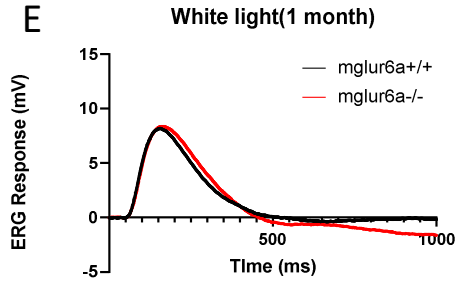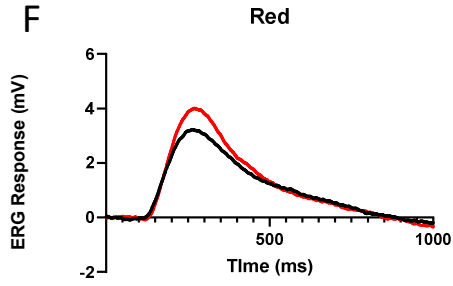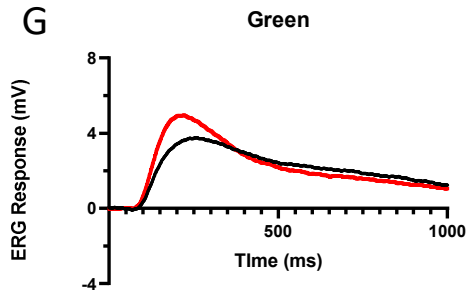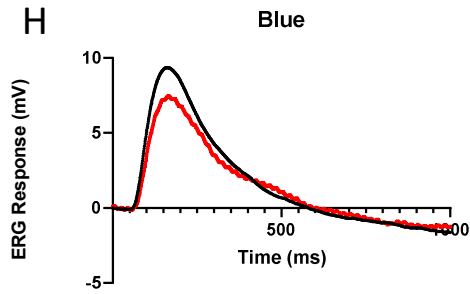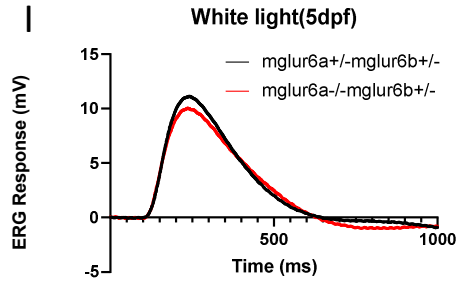

Supplement: Supplement 6 [file iovs-65-12-44_s006.pdf]
