# Supplementary material for: Differential Localization and Functional Roles of mGluR6 Paralogs in Zebrafish Retina
Source: Invest Ophthalmol Vis Sci. 2024 Oct 30;65(12):44. doi: 10.1167/iovs.65.12.44 (PMC11536201; doi:10.1167/iovs.65.12.44)
Supplement: Supplement 7 [file iovs-65-12-44_s007.pdf]

**Amplicon**	**Name**	**Sequence 5'-3'**
**in situ hybridization**		
mglur6a fragment (XY bp)	mglur6a_dr_1345s	CGCGCTGTCAACTTTAATG
	mglur6a_dr_2425as	TGCTGTGCCAAAGAAAATG
mglur6b fragment (XY bp)	mglur6b_dr_-10s	GCCAGCAACTATGACATCAC
	mglur6b_dr_1676as	CATCACAGAGCTCACAATGC
gnaoa fragment (XY bp)	gnaoa_dr_410s	GGCTGGGGAATCTGGGAAAAGTA
	gnaoa_dr_1335as	CAGGTTGTTGGCGATGATGATGTC
	gnaoa_dr_2221as	AGGGGGACAGCGGACGAGAGGTA
gnaob #1	*gnaob_dr_191s*	TGCTGGGCGGAGGGGAGTC
	gnaob_dr_1131as	CAGCCGCGCAGGTTGTTGG (1192)
gnaob #2	*gnaob_dr_193s*	CTGGGCGGAGGGGAGTCG
	gnaob_dr_2058as	AGGAGGAAAACGGGAAAAGAAATC
trpm1a	*trpm1a*_fwd	GCAGGAGAAATGGTCGGT
	*trpm1a*_rev	GGGCGAAGGAAATGATGT
trpm1b fragment (XY bp)	trpm1b_dr_s	AGAGGGCATGGATTGAAAGG
	trpm1b_dr_as	GGTTTGGTAGGGTCGTGT
	trpm1b_dr_as	TCACACACCACCACAGGCA
nyx #1 fragment (XY bp)	nyx_dr_s	GCACATGCACTCAGGAGAAG
	nyx_dr_as	CGATTCTCTTGCAAGTTGAGG
nyx #2 fragment (XY bp)	nyx_dr_s	GGCTTGCACACGCTCCT
	nyx_dr_as	AGTCTGAGAAGCACCGAACA
**CRISPR/Cas9 mutation**		
pT7-gRNA insert	pT7-gRNA_fwd	CAGCTATGACCATGATTACG
	pT7-gRNA_rev	AAAAGCACCGACTCGGTG
pSp6 gRNA insert	pSp6-gRNA_fwd	ATTTAGGTGACACTATA
	pSp6-gRNA_rev	ATTTAGGTGACACTATA
mglur6a crispr target sites	region 4_T7	CGAGGAGGTCCAATCTAACC
	region 5_Sp6	GACCAGGAGGACGTGGCTGA
mglur6a oligonucleotides for generating sgRNA	fwd	CCGCGAGGAGGTCCAATCTAACC
	rev	GACCAGGAGGACGTGGCTGAAGG
mglur6a amplification of target region (258 bp)	mglur6a_dr_Intron3-3s	GCTTGAGCATAAAACTCTAATTC
	mglur6a_dr_Intron4-1as	CAGAGGATGCACATTATATTTC

Supplemental table S1: Primers used for ISH and CrispR/Cas9 genome editing.
