# Supplementary material for: Differential Localization and Functional Roles of mGluR6 Paralogs in Zebrafish Retina
Source: Invest Ophthalmol Vis Sci. 2024 Oct 30;65(12):44. doi: 10.1167/iovs.65.12.44 (PMC11536201; doi:10.1167/iovs.65.12.44)
Supplement: Supplement 8 [file iovs-65-12-44_s008.pdf]

**cone contacts with mGluR6a signal**		n eyes	n total cells
red	0%	2	80
green	63%	1	16
blue	38%	2	16
UV	10%	2	20
rod	100%	2	74
**cone contacts with mGluR6b signal**		n eyes	n total cells
red	61%	6	172
green	100%	2	42
blue	100%	3	47
UV	100%	5	71
rod	100%	2	180

Supplemental table S2: Percentage of cones with mGlur6 paralog puncta overlap.
